# Predicting low cognitive ability at age 5 using machine learning methods and birth cohort data

**DOI:** 10.1093/eurpub/ckac131.422

**Published:** 2022-10-25

**Authors:** A Bowe, A Staines, F McCarthy, G Lightbody, D Murray

**Affiliations:** 1 INFANT Research Centre, University College Cork, Cork, Ireland; 2 Nursing, Psychotherapy and Community Health, Dublin City University, Dublin, Ireland; 3 Paediatrics, Cork University Hospital, Cork, Ireland; 4 Obstetrics and Gynaecology, University College Cork, Cork, Ireland; 5 Electrical and Electronic Engineering, University College Cork, Cork, Ireland

## Abstract

**Background:**

Early intervention is essential to address disparities in cognitive development. Current developmental screening will not detect the vast majority of children who go on to have below average cognitive ability at school age. In this study, we applied the random forest (RF) algorithm, a highly interpretable machine learning method, to birth-cohort data to train a model to predict low cognitive ability at 5 years of age using perinatal features.

**Methods:**

Data was from 1,070 participants in the Irish population-based BASELINE birth cohort. A RF model was trained to predict an intelligence quotient (IQ) score <90 at age 5 years using a broad selection of maternal, infant, birth, and sociodemographic features, all of which could be easily measured at a population level in the perinatal period. Feature importance was examined using mean decrease in Gini impurity, mean decrease in accuracy, and mean minimal depth. Recursive feature elimination was used to develop a parsimonious model. Internal validation was performed using 10-fold cross validation repeated 5 times.

**Results:**

The most predictive features for low cognitive ability at 5 years of age were the total years of maternal schooling, infant Apgar score at 1 minute, socioeconomic index, maternal BMI, and units of alcohol consumed in the first trimester. A parsimonious RF model based on 11 features showed excellent predictive ability, with a sensitivity of 0.89 and a specificity of 0.98, providing a foundation suitable for external validation in an unseen cohort.

**Conclusions:**

Machine learning approaches to large existing datasets can provide accurate feature selection to improve risk prediction. Further validation of this model is required in additional cohorts, representative of the general population. Accurate risk prediction can facilitate targeted screening and intervention.

**Key messages:**

• The application of machine learning to large population-based data can improve feature selection and accuracy in risk prediction models.

• Accurate risk prediction may enable early intervention to address disparities in cognitive development. Individual interventions must occur in conjunction with population level policy changes.

